# West Nile Virus: High Transmission Rate in North-Western European Mosquitoes Indicates Its Epidemic Potential and Warrants Increased Surveillance

**DOI:** 10.1371/journal.pntd.0003956

**Published:** 2015-07-30

**Authors:** Jelke J. Fros, Corinne Geertsema, Chantal B. Vogels, Peter P. Roosjen, Anna-Bella Failloux, Just M. Vlak, Constantianus J. Koenraadt, Willem Takken, Gorben P. Pijlman

**Affiliations:** 1 Laboratory of Virology, Wageningen University, Wageningen, The Netherlands; 2 Laboratory of Entomology, Wageningen University, Wageningen, The Netherlands; 3 Laboratory of Geo-information Science and Remote Sensing, Wageningen University, Wageningen, The Netherlands; 4 Institut Pasteur, Department of Virology, Arboviruses and Insect Vectors, Paris, France; University of Florida, UNITED STATES

## Abstract

**Background:**

West Nile virus (WNV) is a highly pathogenic flavivirus transmitted by *Culex spp*. mosquitoes. In North America (NA), lineage 1 WNV caused the largest outbreak of neuroinvasive disease to date, while a novel pathogenic lineage 2 strain circulates in southern Europe. To estimate WNV lineage 2 epidemic potential it is paramount to know if mosquitoes from currently WNV-free areas can support further spread of this epidemic.

**Methodology/Principal Findings:**

We assessed WNV vector competence of *Culex pipiens* mosquitoes originating from north-western Europe (NWE) in direct comparison with those from NA. We exposed mosquitoes to infectious blood meals of lineage 1 or 2 WNV and determined the infection and transmission rates. We explored reasons for vector competence differences by comparing intrathoracic injection versus blood meal infection, and we investigated the influence of temperature. We found that NWE mosquitoes are highly competent for both WNV lineages, with transmission rates up to 25%. Compared to NA mosquitoes, transmission rates for lineage 2 WNV were significantly elevated in NWE mosquitoes due to better virus dissemination from the midgut and a shorter extrinsic incubation time. WNV infection rates further increased with temperature increase.

**Conclusions/Significance:**

Our study provides experimental evidence to indicate markedly different risk levels between both continents for lineage 2 WNV transmission and suggests a degree of genotype-genotype specificity in the interaction between virus and vector. Our experiments with varying temperatures explain the current localized WNV activity in southern Europe, yet imply further epidemic spread throughout NWE during periods with favourable climatic conditions. This emphasizes the need for intensified surveillance of virus activity in current WNV disease-free regions and warrants increased awareness in clinics throughout Europe.

## Author Summary

West Nile virus (WNV) is on the rise in Europe, with increasing numbers of human cases of neurological disease and death since 2010. However, it is currently unknown whether or not WNV will continue to spread to north-western Europe (NWE), in a fashion similar to the WNV epidemic sweep in the United States (1999–2004). The presence of competent mosquitoes is a strict requirement for WNV transmission, but no laboratory studies have been conducted with the new European lineage 2 WNV outbreak strain. Our study is the first to investigate transmissibility in NWE *Culex pipiens* for lineage 2 WNV in a systematic, direct comparison with North American *Culex pipiens* and with the lineage 1 WNV strain. We demonstrate that European mosquitoes are highly competent for both WNV lineages, which underscores the epidemic potential of WNV in Europe. However, the transmission rate for lineage 2 WNV was significantly lower in North American mosquitoes, which indicates different risk levels between both continents for lineage 2 but not lineage 1 WNV. Based on our result, we propose that WNV surveillance in mosquitoes and birds must be intensified in Europe to allow early detection, timely intervention strategies and prevent outbreaks of WNV neurological disease.

## Introduction

West Nile virus (WNV; family *Flaviviridae*, genus *Flavivirus*) is an important mosquito-borne human pathogen associated with febrile illness, which may develop into severe neuroinvasive disease and death [1]. The pathogenic isolates of WNV can be classified into two lineages. Lineage 1 WNV strains have long been endemic in Africa, Australia, the Middle East, Asia and southern Europe [2,3]. In the 1990s, lineage 1 WNV re-emerged in southern Europe and the Middle-East [4–6]. In 1999, lineage 1 WNV was unintentionally introduced into New York City from where it spread rapidly across the United States where it is now endemic [7]. With an accumulated 17,463 cases of neuroinvasive disease and 1,668 reported deaths between 1999 and 2013, this outbreak quickly evolved into the largest outbreak of neuroinvasive disease to date [8]. Lineage 2 WNV strains have been endemic in sub-Saharan Africa and Madagascar and were previously considered to be of low pathogenicity [2,3]. In 2010, a highly pathogenic lineage 2 WNV isolate caused a large outbreak in Greece [9], which resulted in 262 cases of human disease and 35 deaths. Lineage 2 WNV then quickly became endemic in South-East Europe and with annual outbreaks to date WNV disease in the region has increased seven-fold [10–12]. At present, WNV disease does not extend into north-western Europe (NWE) [11].

During enzootic transmission, WNV circulates primarily between mosquitoes of the *Culex* genus and birds. Many avian species in North America (NA) [13] and Europe [14,15] are suitable reservoirs/amplifying hosts and can produce high viral titres upon WNV infection. Infected mosquitoes also blood feed on other vertebrate hosts, which leads to frequent infections in humans and horses [16]. In Europe, the main *Culex* species found positive during WNV surveys is the common house mosquito *Culex pipiens* [17]. In NA the most prevalent and effective vector species for WNV are *Culex pipiens*, *Culex tarsalis* and *Culex quinquefasciatus* [18,19]. Laboratory experiments show that NA *Culex pipiens* mosquitoes are competent vectors for NA isolates of lineage 1 WNV [20]. The vector competence of European mosquitoes to lineage 1 WNV has not been intensively studied nor has it been compared directly to competent vectors from NA [21]. The vector competence of NA and European mosquitoes for transmission of novel European lineage 2 WNV isolates has not yet been determined, but this is of high importance now that a highly pathogenic lineage 2 WNV has emerged in Europe, which appears to be as neuroinvasive as WNV isolates from lineage 1 [9].

As the global activity of these pathogenic WNV lineages has significantly increased over the past two decades, we set out to assess the potential for virus transmission in areas that are currently free of lineage 1 and/or lineage 2 WNV strains. The results show that European mosquitoes from an area free of WNV disease have the intrinsic capability to transmit both lineage 1 and lineage 2 WNV. However, comparing transmission rates at varying temperatures provides evidence that the differences in climatic conditions currently restrict the spread of WNV throughout Europe.

## Materials and Methods

### Cells and viruses

C6/36 (ATCC CRL-1660) and *Culex tarsalis* cells (CDC, Fort Collins, CO) were grown on Leibovitz L15 and Schneiders (Gibco) medium supplemented with 10% fetal bovine serum (FBS; Gibco). Hela (ATCC CCL-2), DF-1 (ATCC CRL-12203) and Vero E6 (ATCC CRL-1586) cells were cultured with DMEM Hepes (Gibco, Bleiswijk, The Netherlands) buffered medium supplemented with 10% FBS containing penicillin (100IU/ml) and streptomycin (100μg/ml). When Vero E6 cells were incubated with mosquito lysates or saliva the growth medium was supplemented with fungizone (2,5μg/ml) and gentamycin (50μg/ml). P2 virus stocks of the NY’99 and Gr’10 isolates were grown on C6/36 cells and titrated on Vero E6 cells.

### Mosquito rearing

The NWE *Culex pipiens* colony originated from Brummen, The Netherlands (°05'23.2"N 6°09'20.1"E) and was established in 2010 and maintained at 23°C. The NA *Culex pipiens* colony [20] was maintained at 26°C. Both mosquito colonies were kept in Bugdorm cages with a 16:8 light:dark (L:D) cycle and 60% relative humidity (RH) and were provided with 6% glucose solution. Bovine or chicken whole blood (KemperKip, Uden, The Netherlands) was provided through the Hemotek PS5 (Discovery Workshops) for egg production. Egg rafts were allowed to hatch in tap water supplemented with Liquifry No. 1 (Interpet Ltd., Dorking, UK). Larvae were fed with a 1:1:1 mixture of bovine liver powder (Sigma-Aldrich, Zwijndrecht, The Netherlands), ground rabbit food and ground koi food.

### *In vivo* infections

2–5 day old mosquitoes were infected either via ingestion of an infectious blood meal or via intrathoracic injections. Infectious blood meals: Whole chicken blood was mixed with the respective P2 virus stock to a final concentration of 1.4*10^8^ WNV infectious particles per ml. Mosquitoes were allowed to membrane feed, using the Hemotek system and a parafilm membrane, in a dark climate controlled room (24°C, 70% RH). After 1 hour, mosquitoes were sedated with 100% CO_2_ and the fully engorged females were selected. Injections: Mosquitoes were sedated with CO_2_ and placed on a semi-permeable pad, attached to 100% CO_2_. Mosquitoes were infected by intrathoracic injection using the Drummond nanoject 2 (Drummond scientific company, United States). Infected mosquitoes were incubated at their respective temperatures with a 16:8 L:D cycle and fed with 6% sugar water during the course of the experiment.

### Salivation assay

Legs and wings of sedated mosquitoes were removed and their proboscis was inserted into a 200ul filter tip containing 5 ul of salivation medium (50% FBS and 50% sugar water (glucose, W/V 50%)). Mosquitoes were allowed to salivate for 45 minutes. Mosquito bodies were frozen in individual Eppendorf tubes containing 0.5 mm zirconium beads (Next Advance, New York, USA) at -80°C. The mixture containing the saliva was added to 55 ul of fully supplemented growth medium.

### WNV infectivity assay

Frozen mosquito bodies were homogenized in the bullet blender storm (Next Advance New York, USA) in 100 μl of fully supplemented medium and centrifuged for 90 s at 14000 rpm in a table top centrifuge. 30ul of the supernatant from the mosquito homogenate or the saliva containing mixture was incubated on a monolayer of Vero cells in a 96-wells plate. After 2–4 hours the medium was replaced by 100 μl of fresh fully supplemented medium. Wells were scored for WNV-specific cytopathic effects (CPE), confirmed with immunofluorescence assay (IFA) against WNV E [22] at three days post infection (dpi). WNV titres were determined using 10μl of the supernatant from the mosquito homogenate in end point dilution assays on Vero E6 cells. WNV infection was scored by CPE, confirmed with IFA at three dpi.

### Temperature maps

Maps displaying the mean diurnal temperature during July and August of the indicated year [23]. Human cases of WNV in Europe, during 2011, 2012 or 2013 were projected on the location where they were reported [11]. To eliminate potential imported cases, WNV cases were only considered when a country reported more than one case for that year.

### Statistical analysis

WNV infections in mosquito bodies and saliva were scored positive or negative and significant differences were calculated using the Fisher’s exact test (P<0.05). Differences in WNV titres (TCID50/ml) in infected mosquito bodies and heads were calculated using the Mann Whitney test (P<0.05).

## Results

### Transmission rate of lineage 2 WNV is higher in European than American mosquitoes

**Fig 1:**
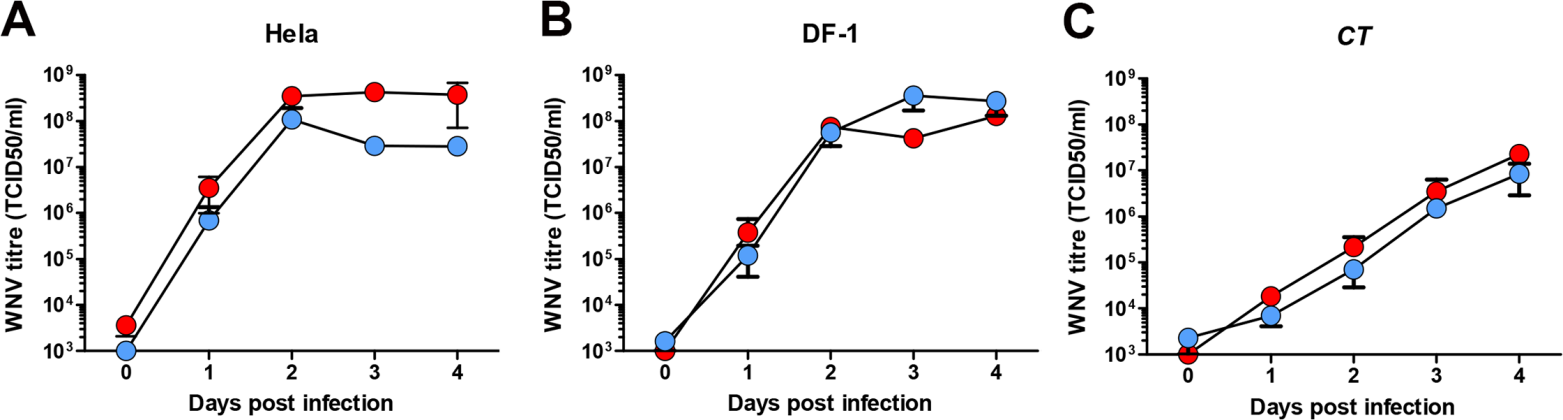
Growth kinetics of lineage 1 and 2 WNV strains. (A) Human Hela, (B) avian DF-1 (duck fibroblasts) and (C) mosquito *CT* (*Culex tarsalis*) cells infected with WNV-lin2 (red) or WNV-lin1 (blue) isolates at a multiplicity of infection of 1. Medium was harvested at the indicated days post infection and used in end point dilution assays. Error bars represent the standard error of the mean.

**Fig 2:**
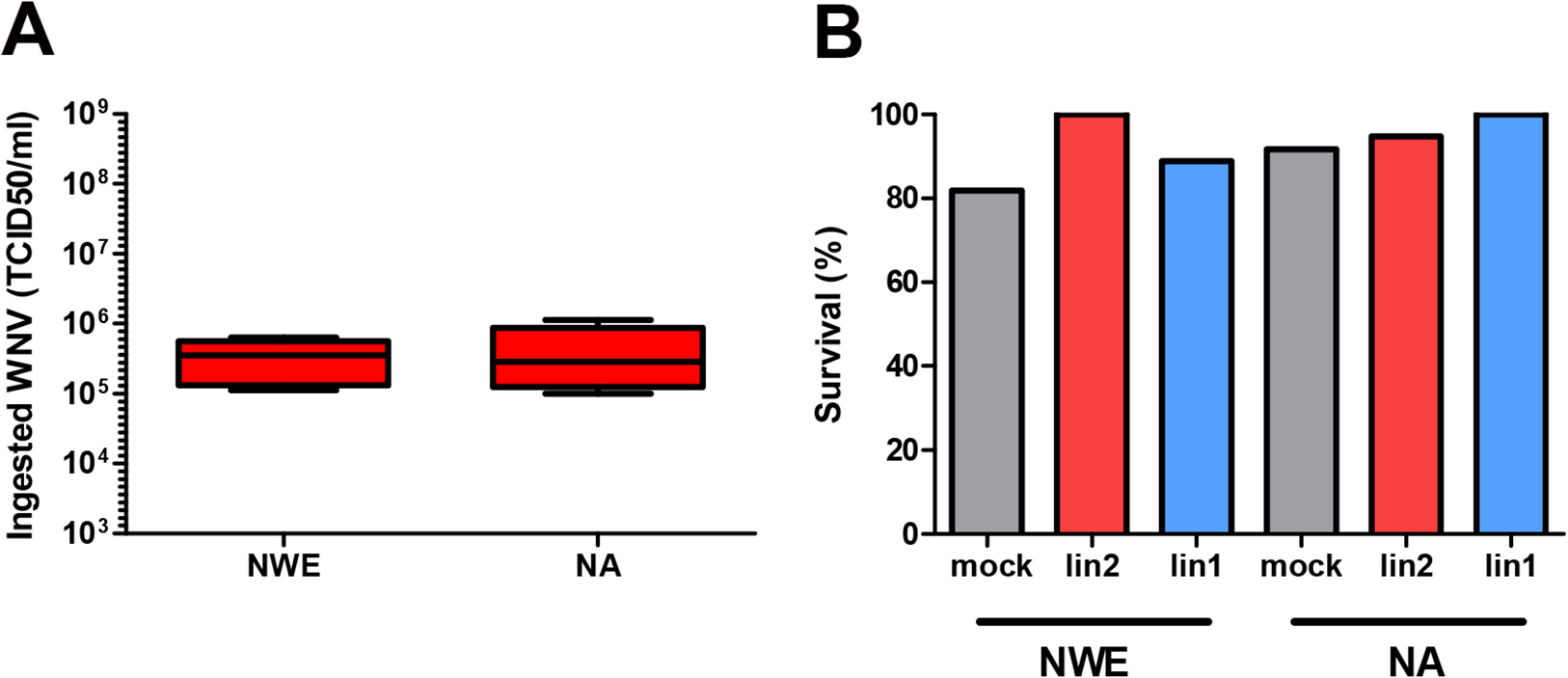
Both NWE and NA *Culex pipiens* mosquitoes ingest equal amounts of infectious virus with identical survival rates. (A) Mosquitoes were infected with WNV-lin2 via an infectious blood meal, homogenized, and the viral titres were determined in end point dilution assays. Results are represented as a Tukey box plot. (B) Mosquitoes were offered a non-infectious blood meal (mock) or a blood meal containing either WNV isolate. Bars represent the percentage of surviving mosquitoes at 14 days post infection.

**Fig 3:**
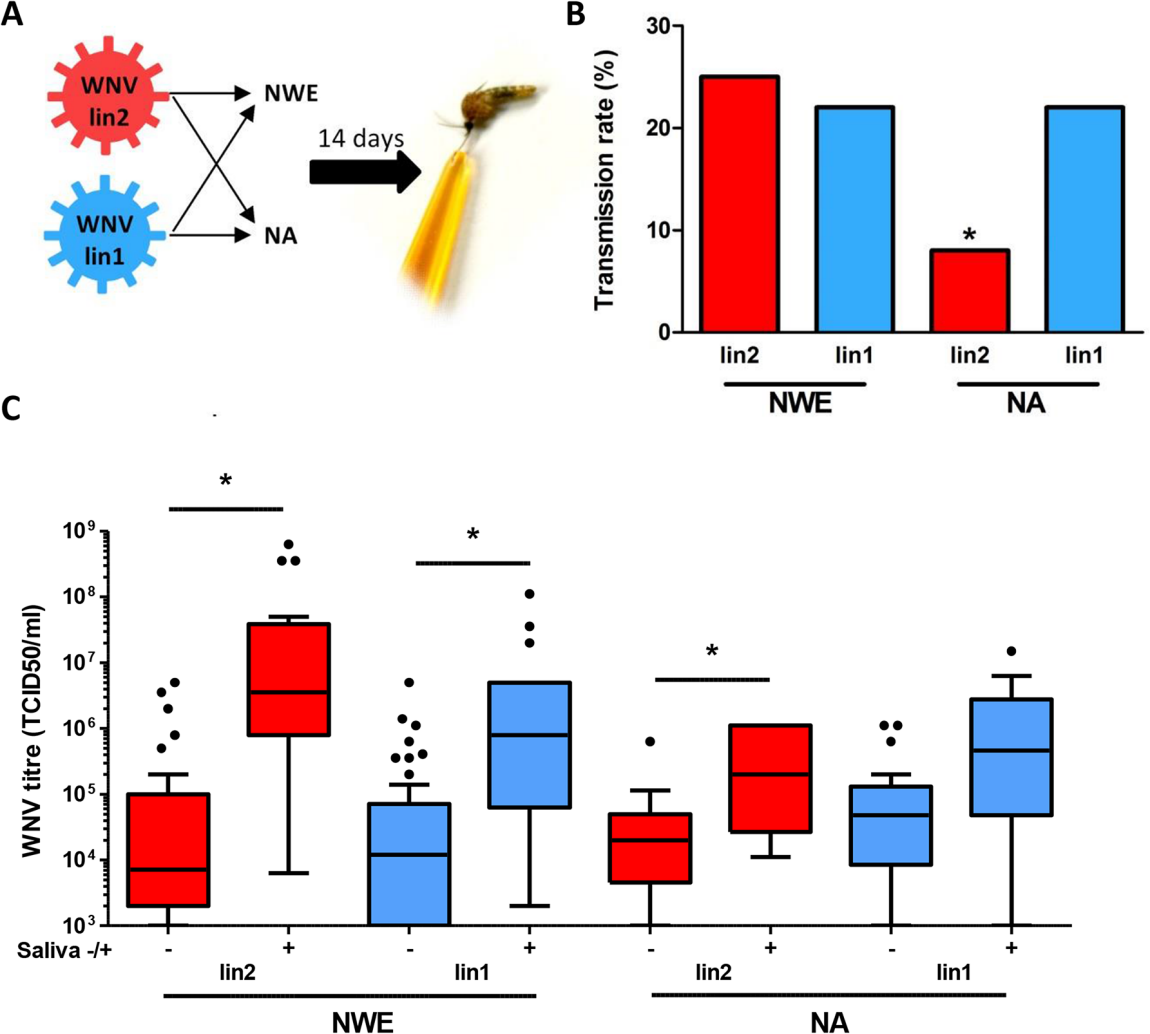
European *Culex pipiens* mosquitoes are competent vectors for pathogenic WNV lineages 1 and 2. (A) Schematic representation of the experiment. Mosquitoes from NWE or NA were given a blood meal containing virus from either WNV-lin2 or WNV-lin1 isolates. (B) Fourteen days post infection the saliva was harvested and scored for infectious WNV. Bars represent the percentage of mosquitoes with infectious saliva. (C) Ten μl of the homogenized mosquito bodies was titrated in end point dilution assays. For each sample population, the WNV titres of individual mosquito bodies were grouped into saliva negative (left) and positive (right) populations, represented in Tukey box plots. Individual outliers are indicated. Asterisks indicate significant differences (P<0.05, Fisher’s exact test (B), Man Whitney test (C)).

*Culex pipiens* mosquitoes from NWE (The Netherlands), and a NA *Culex pipiens* colony [20] were infected with either the novel pathogenic lineage 2 WNV isolate (WNV-lin2) from Greece’10 or lineage 1 isolate (WNV-lin1), New York ‘99. The vector competence of NA mosquitoes for WNV-lin1 has been well-described [20] and serves as a reference for the infection and transmission rates of WNV. The WNV-lin2 and WNV-lin1 isolates displayed similar growth kinetics in human, avian and mosquito cell cultures (Fig 1A–1C). Infectious blood meals containing 1.4*10^8^ TCID_50_/ml of either WNV-lin2 or WNV-lin1 isolates were fed to the NWE and NA *Culex pipiens* mosquitoes. Fully engorged females were selected and kept at an ambient temperature of 23°C. Immediately after completion of the blood meal, a subset of fully engorged females was tested for the presence of infectious WNV to confirm that both mosquito populations had ingested equal amounts of infectious virus particles (Fig 2A). Infection with either WNV isolate did not influence mosquito survival during the course of the experiments (Fig 2B). After 14 days, saliva was collected from a large and random subset of the mosquitoes. Both the isolated saliva as well as all the mosquito bodies were examined for the presence of WNV (schematic representation of the experiment, Fig 3A). The combined results from five independent experiments are summarized in Table 1 (infection rates, bodies) and 2 (transmission rates, saliva). Both the NWE and NA mosquitoes were equally susceptible to infection, but with significant differences in the infection rates between the WNV-lin2 and WNV-lin1 isolates (Table 1, P<0.05).

**Table 1:** Infection rate of lineage 1 and 2 WNV isolates in NWE and NA *Culex pipiens* mosquitoes.

Mosquito population	WNV lineage	n (#)	Infected bodies (#)	Infection rate (%)
NWE	lin2	154	51	33
NA	lin2	102	29	28
NWE	lin1	131	64	49
NA	lin1	87	36	42

Dissemination of WNV into the saliva of a vector is a prerequisite for successful transmission. After consuming a blood meal that contained WNV-lin1, 22% and 19% of respectively the NWE and NA mosquitoes had detectable levels of WNV in their saliva (Fig 3B). In contrast, the WNV-lin2 isolate was detectable in the saliva of 24% of the NWE mosquitoes, but only in 8% of the NA mosquitoes (Fig 3B, P<0.05) indicative of a strongly reduced susceptibility of the latter for WNV-lin2. The observed differences in transmission rates between WNV-lin2 infected NWE and NA mosquitoes are underscored when only the percentage of WNV-infected mosquitoes are considered. From the population of WNV-infected mosquitoes, successful replication and dissemination of WNV-lin2 into the saliva was found in 59% of the NWE, compared to only 24% of the NA mosquitoes (Table 2).

**Table 2:** Transmission rate of lineage 1 and 2 WNV isolates in NWE and NA *Culex pipiens* mosquitoes.

Mosquito population	WNV lineage	n (#)	Infected bodies (#)	Positive saliva (#)	Transmission rate (%)	Positive saliva (% of positive mosquitoes)
NWE	lin2	79	32	19	24	59
NA	lin2	102	29	7	8	24
NWE	lin1	67	40	15	22	38
NA	lin1	74	33	14	19	42

In an effort to understand these differences in transmissibility we first determined the tissue culture infectious dose of WNV present in each positive mosquito body by end point dilution assays. The viral titres in individual mosquito bodies were highly variable and could reach up to 10^9^ TCID_50_/ml for WNV-lin2 infected NWE mosquitoes, compared to a three logs lower maximum titre of only 10^6^ TCID_50_/ml in NA mosquitoes (Fig 3C). Comparison between the WNV titres of saliva-positive and saliva-negative mosquitoes within the same sample population showed that significantly more infectious WNV particles were present in the bodies of mosquitoes with positive saliva than with negative saliva (Fig 3C). This indicates that the level of WNV replication in the mosquito body determines the dissemination into the salivary glands.

### Differential transmission rates of WNV-lin2 attributed to infection barriers

**Fig 4:**
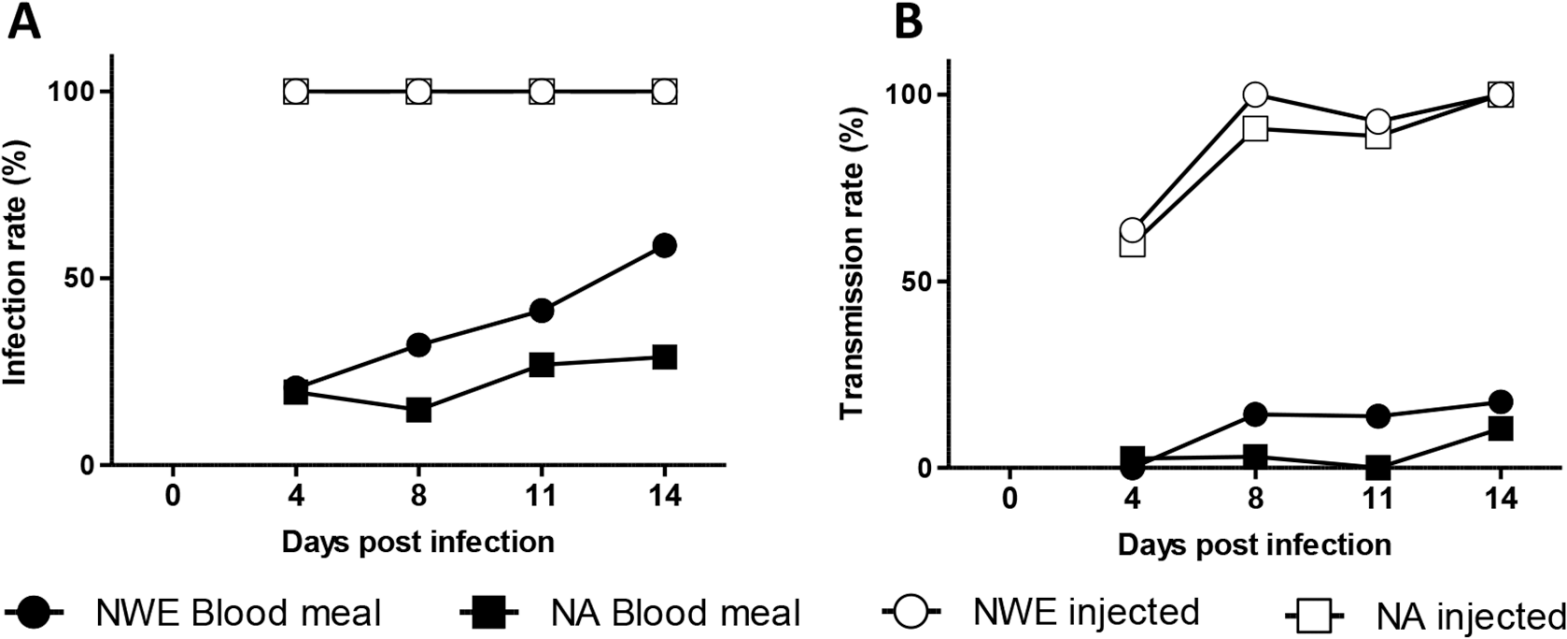
Mesenteron infection barriers determine the transmission rate of WNV-lin2. *Culex pipiens* from either NWE (spherical symbols) or NA (square symbols) were infected with the WNV-lin2 isolate via infectious blood meals (closed symbols) or intrathoracic injections (open symbols). At the indicated times post infection, the percentages of effectively infected mosquitoes (A) or successive infectious saliva (B) were determined.

Efficient infection and escape from the midgut epithelial cells is necessary for dissemination of the virus to other tissues, including the salivary glands [24–26]. When the midgut was circumvented by injecting WNV-lin2 directly into the thorax, all mosquitoes from both NWE and NA became readily infected (Fig 4A, open symbols) and up to 100% of injected individuals were able to transmit WNV at day eight post injection (Fig 4B, open symbols). In contrast, infectious blood meals resulted in differential proportions of the NWE and NA mosquitoes being able to transmit WNV-lin2 (Fig 4B, closed symbols), again with NWE as a more competent vector. Strikingly, both eight and eleven days post infection, the WNV-lin2 isolate was detected in the saliva of 14% of NWE mosquitoes, compared to <3% of NA mosquitoes (Fig 4B, closed symbols, P = 0.1076 and P = 0.0259 respectively).

Thus, oral infection with the WNV-lin2 isolate results in better dissemination and a shorter mean extrinsic incubation period, suggesting that WNV-lin2 escapes more effectively from the midgut epithelial cells in mosquitoes from NWE compared to those from NA. Taken together, transmission of both WNV lineages is intrinsically possible in NWE whereas there is no evidence to suggest that WNV-lin2 can utilize NA mosquitoes as effective vectors due to limited dissemination to the salivary glands.

### Higher temperatures increase WNV infection rate

**Fig 5:**
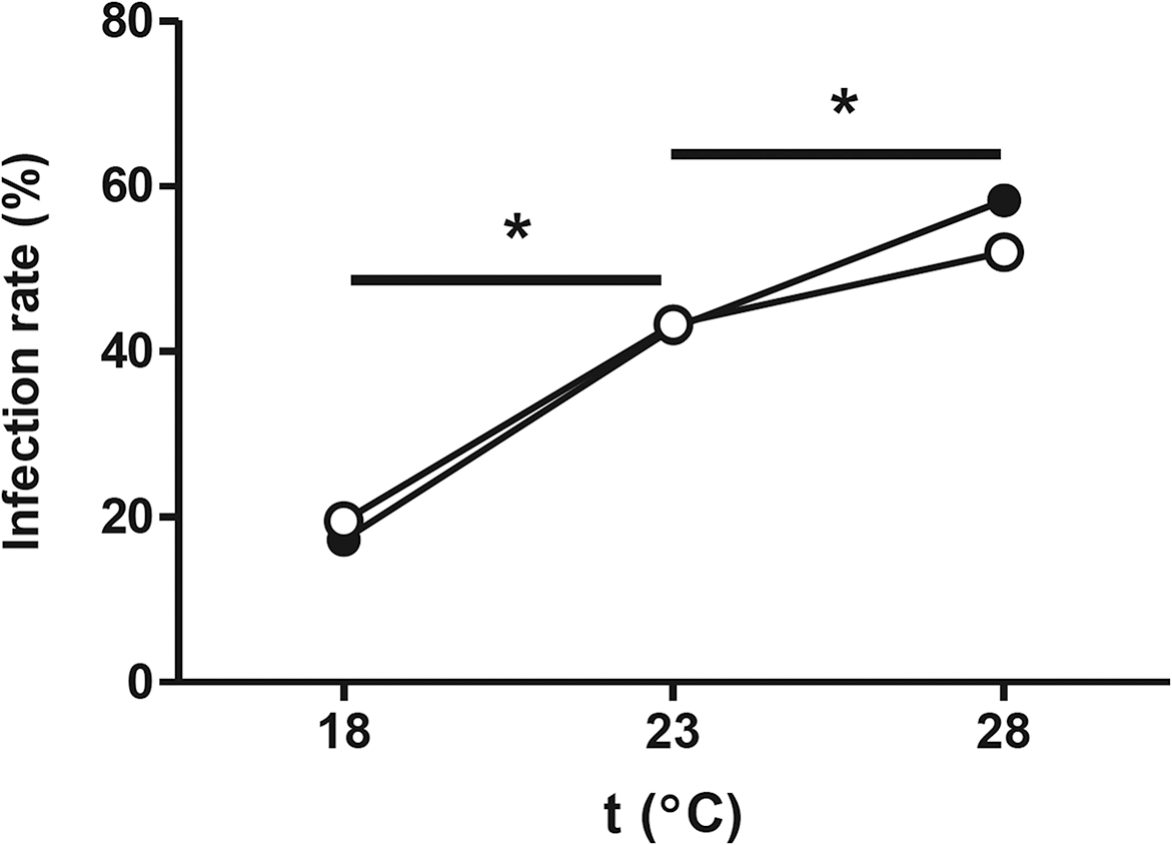
Higher temperatures increase WNV infection rate in *Culex pipiens*. Both NA (open symbols) and NWE (closed symbols) mosquitoes were orally infected with the WNV-lin2 isolate via a blood meal. Engorged mosquitoes were separated into three groups which were incubated at either 18, 23 or 28°C. The infection rate was determined 14 days post oral infections. Asterisks indicate significant differences (Fisher’s exact test P<0.05).

As the mosquito colonies used in this laboratory study are representatives of their respective populations from the described areas, the experiments presented here show that highly WNV-competent *Culex pipiens* mosquitoes are present in NWE. Vector competence is, however, not only attributed to intrinsic factors, but also subjective to extrinsic factors, most notably the ambient temperature [20,27]. Because indigenous WNV activity is currently absent in NWE [11,28], but competent European bird species are present [14,15], we hypothesized that temperature limits the vector competence of European mosquitoes for WNV transmission. To test this hypothesis, we infected both NWE and NA mosquitoes with the WNV-lin2 isolate via a WNV-containing blood meal and incubated the mosquitoes at three different temperatures for 14 days post oral infections. The first temperature represented the average summer temperatures in large parts of NWE, including the origin of our NWE mosquito colony (The Netherlands; 18°C). The second temperature was an intermediate temperature (23°C), while the third temperature matched the average summer temperature of the area where WNV-lin2 was isolated (Greece; 28°C) [29]. The warmest period of the year (July and August) also corresponded with the peak in WNV amplification and transmission [8]. Higher temperatures significantly increased the percentage of WNV-infected mosquito vectors, with no apparent difference between NWE and NA mosquitoes (Fig 5, P<0.05). At 18°C, 17% (n = 29) and 19% (n = 41) of mosquitoes were infected with WNV-lin2, whereas incubation at 28°C increased the infection rates to 58% (n = 36) and 52% (n = 25) for NWE and NA mosquitoes, respectively.

**Fig 6:**
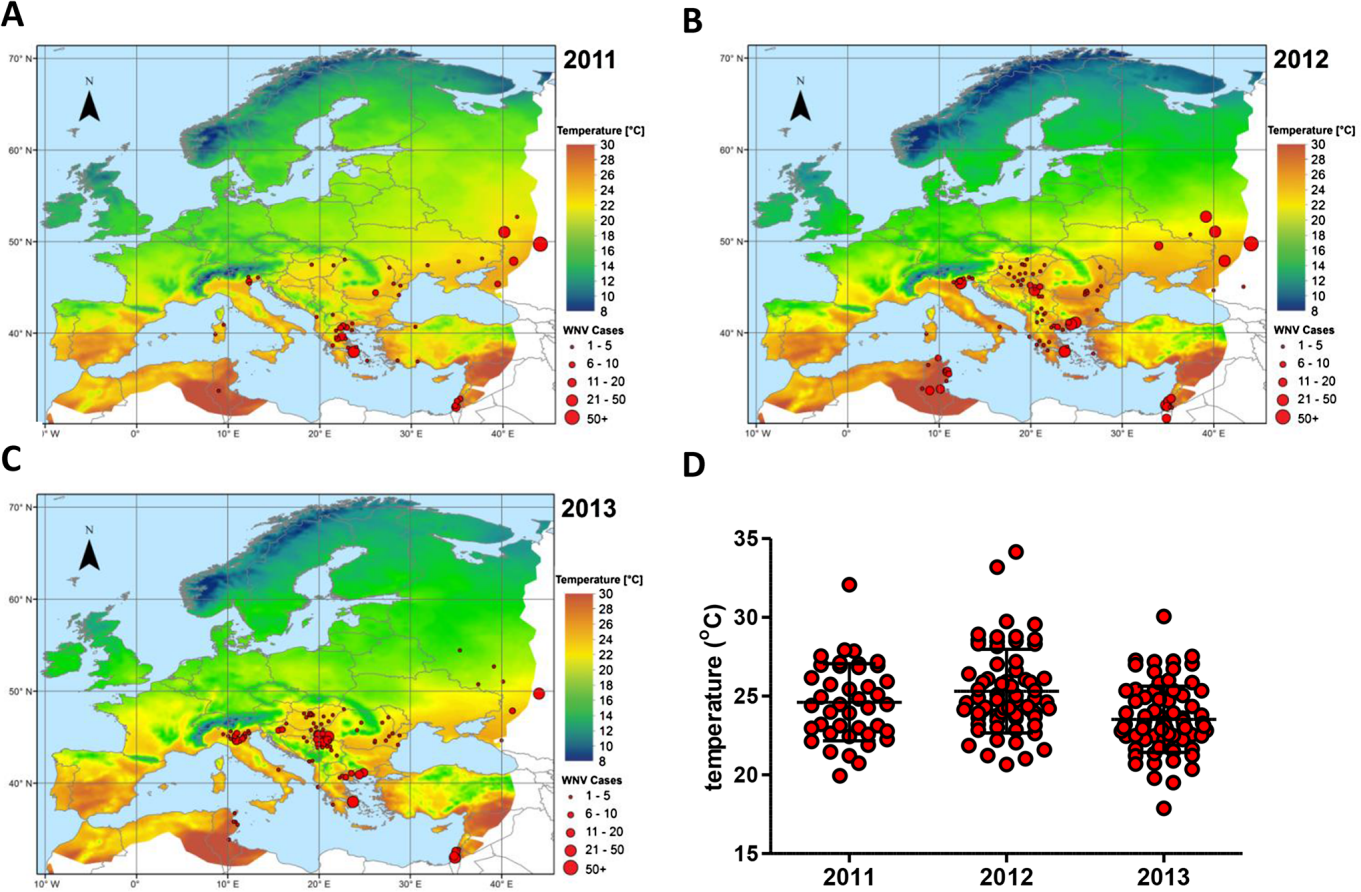
Mean diurnal summer temperature correlates with European WNV outbreaks. Mean diurnal temperature during July-August of (A) 2011, (B) 2012 and (C) 2013. Dots represent human WNV cases reported in the respective year. (D) Scatter plot displays the mean temperature during July and August of the indicated year at each individual location with WNV activity. The mean temperatures and standard deviations are indicated.

Comparison between the spatial arrangement of recent WNV outbreaks in Europe per annum and the corresponding mean temperature during peak transmission season strengthens this hypothesis by displaying a strong correlation between WNV outbreaks and the mean diurnal summer temperature throughout Europe (Fig 6A and 6B and 6C). The mean temperatures at which WNV outbreaks occurred in 2011, 2012 and 2013 were 24.6°C, 25.3°C, and 23.5°C, with standard deviations of 2.4°C, 2.7°C, and 2.1°C, respectively (Fig 6D). Together, the mean temperatures at the respective locations of individual outbreaks give an indication of the average summer temperatures at which there is an elevated risk for WNV activity.

## Discussion

Here we show that both pathogenic lineages of WNV can effectively infect mosquitoes from NWE. Our finding that two geographically separated *Culex pipiens* populations (NWE and NA) have a markedly different vector competence for WNV-lin2, suggests a degree of genotype-genotype specificity in the interaction between virus and vector. Alternatively, the presence of certain endosymbionts or insect-specific flaviviruses can have an effect on the vector competence as well [30,31]. As the differential infection rate is only apparent when WNV is infected orally and not via intrathoracic injections, this suggests that WNV-lin2 escapes more effectively from the midgut epithelial cells in mosquitoes from NWE compared to those from NA.

The presence of highly competent vector species in WNV disease-free areas suggests that extrinsic factors such as temperature play an essential role in the current distribution of WNV. In NWE, the lower average summer temperature (<20°C) may provide a possible explanation for the current WNV epidemics, which remain restricted to southern Europe.

However, other extrinsic factors can shape the vectorial capacity and may compensate for a reduced vector competence at low temperature by facilitating larger mosquito populations. The recent resurgence of WNV disease in the United States was most likely fuelled by climatic conditions that were favourable for local vector populations [32,33]. In addition, hybrids between two closely related *Culex pipiens* forms may increase the incidence of human WNV disease, as these ‘bridge-vectors’ are considered less ornithophilic and more likely to feed on other vertebrates, including humans. These hybrids are relatively common in North America, but not in north-western Europe [34]. How effective different European *Culex pipiens* populations are in transmitting WNV is currently unknown, but this can be investigated by using wild caught *Culex pipiens* mosquitoes from a variety of sources and regions.

Additionally, viral adaptations that increase the replication efficiency at lower temperatures could further facilitate and enhance transmission of WNV throughout Europe. Indeed, the WNV-lin1 isolate has already proven to be able to adapt to the different climatic conditions in the Americas [20], while other flaviviruses, including a close relative of WNV, Usutu virus, are already endemic in parts of NWE [35].

Finally, travel and trade continuously (re-)introduce WNV to areas free of overt WNV-disease. In addition, WNV transmission in the absence of noticeable disease has been suggested based upon serological surveys in (sentinel) birds [36, 37], which may spark a WNV epidemic in NWE. The presence of vectors that are intrinsically capable of transmitting WNV increases the chances for novel outbreaks of WNV-disease, especially when global warming or temporary weather extremes will favour the vectorial capacity of *Culex pipiens*. Based on our results and experimental evidence by others that European birds are suitable amplifying hosts [14], we propose that WNV surveillance in mosquitoes and birds should be intensified, especially in areas where climatic conditions are more favourable, to allow early detection and the implementation of effective mitigation and intervention strategies. Furthermore, awareness by clinicians throughout Europe is warranted in order to more effectively diagnose cases of human WNV (neurological) disease.
